# Letter to the Editor: Novel TREM2 frameshift mutation in a 30-year-old woman with suspected frontotemporal dementia

**DOI:** 10.1007/s10072-023-06726-8

**Published:** 2023-03-10

**Authors:** Maria Buthut, Philipp Reber, Eberhard Siebert, Katharina Eisenhut, Franziska Thaler, Josefine Finck, Surjo R. Soekadar, Harald Prüss

**Affiliations:** 1 German Center for Neurodegenerative Diseases (DZNE) Berlin, Berlin, Germany; 2 Department of Psychiatry and Neurosciences, Clinical Neurotechnology Laboratory, Neuroscience Research Center, Campus Charité Mitte (CCM), Charité – Universitätsmedizin Berlin, Berlin, Germany; 3 Department of Neurology and Experimental Neurology, Charité – Universitätsmedizin Berlin, Corporate Member of Freie Universität Berlin, Humboldt-Universität Berlin, and Berlin Institute of Health, Berlin, Germany; 4 Department of Neuroradiology, Charité – Universitätsmedizin Berlin, Berlin, Germany; 5 Institute of Clinical Neuroimmunology, University Hospital and Biomedical Center, Ludwig-Maximilians-Universität, Munich, Germany; 6 Munich Cluster for Systems Neurology (SyNergy), Munich, Germany

Dear Editor in Chief,

We here report the case of a young woman with suspected behavioural variant frontotemporal dementia (bvFTD) including progressive cognitive decline, mood swings and impaired impulse control that strongly supports early implementation of extensive diagnostics in unclear cases with complex neuropsychiatric symptoms.

A 30-year-old patient was referred to our outpatient memory clinic with suspected diagnosis of bvFTD for further evaluation. She and her mother reported a completely inconspicuous development until the age of 28 with a high school diploma and bachelor’s degree “A” three years ago and negative family history for neuropsychiatric diseases. Around her early twenties she had once broken her left ankle by falling off a horse, which led to surgery. Orthopaedics described bone cysts in the talus in computed tomography (CT) scans (Fig. 1E + F). Further imaging work-up using magnetic resonance imaging (MRI) (Fig. 1G) revealed three bone cysts within the talus and fibular malleolus with central fat signal intensity. However, no specific diagnosis was given at that time, although the patient reported episodes of joint pain of the knees, ankles, and wrists since adolescence.

**Fig. 1:**
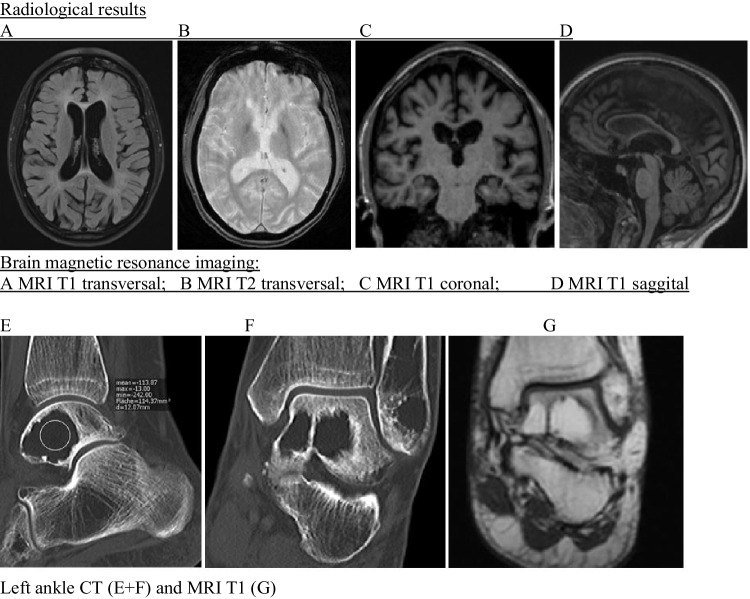
Radiological results

Shortly after starting to work on her master`s degree, the first behavioural changes occurred. She quit university, broke up a long-term relationship, moved and focused on risk sports. Due to a progressive lack of motivation, mood swings and verbal aggressive behaviour, she presented to a psychiatric outpatient department and was diagnosed of a depressive episode and adjustment disorder. The mother further noticed progressive cognitive decline with impaired concentration during the last two years. Basic blood tests were unremarkable, MRI of the brain showed supratentorial cortical and subcortical atrophy with mild periventricular leukoencephalopathy and relatively preserved infratentorial structures as well as subtle bilateral calcifications of the globi pallidi (Fig. 1A–D).

We initiated extended neuropsychological testing (including TAP, WMS-R, VLMT-A, ROCF-A, MWT-A, LPS, Stroop, BDI-II, RWT, BNT) which revealed impairment of attention, executive and memory functions (Table 1). The patient herself had complete anosognosia, we noted discrete disinhibition, euphoria, *Witzelsucht*, logorrhoea and further behavioural symptoms including disturbed impulse control, predominantly increased eating and increased distractibility. Disinhibition particularly increased during the following course of disease and led to significant weight gain as well as increased interest in random sexual contacts. Her speech was fluent and coherent most of the time. There were no abnormalities in motor examination, reflexes were symmetrically brisk and primitive reflexes were absent.

**Table 1:** Paraclinical results

CSF		Blood		Neuropsychological tests	
Cell count (number/µl)	1 [≤ 4]	CRP (mg/l)	1.8 [< 5]	Verbal/semantic word fluency	PR 3
protein (mg/l)	406.9 [150–450]	TSH (mU/l)	1.89 [0.27–4.2]	RCFT *rey complex figure test*	**PR < 1**
Oligoclonal bands	neg	Oligoclonal bands	neg	VLMT-A *verbal learn and memory test*	PR 35–60
Tau (pg/ml)	336 [< 451]	Sodium (mmol/l)	141 [136–145]	Attention(spared/simple)	**PR 8**
pTau181 (pg/ml)	27 [< 62]	Potassium (mmol/l)	4.1 [3.4–4.5]	MWT-A *multiple choice vocabulary intelligence test*	PR 84
Beta-amyloid-ratio	0.115 [> 0.095]	Parathyroid hormone (ng/l)	34.4 [15–65]	LPS *intelligence test*	PR 31
Nfl (pg/ml)	**2305** [< 379]	Osteocalcin (ng/ml)	20.2 [8.4–34.8]	Stroop *color-word-interference test*	PR 16
Antineuronal autoantibodies*	neg	Antineuronal autoantibodies*	Neg	BDI-II *Beck depression inventory*	8
sTREM2** (pg/ml) NHD-patient	**0.0**	sTREM2 (pg/ml) NHD-patient	**30.76**		
sTREM2** (pg/ml) healthy control	915.44	sTREM2 (pg/ml) Healthy control	130.42		
sTREM2** (pg/ml) Alzheimer’s control	3179.69	sTREM2 (pg/ml) Alzheimer’s control	499.22		

Results in bolt are pathological

^*^Tested antineuronal autoantibodies: Aquaporin-4, Glutamate receptors (type NMDA), GABA-b-receptors, CASPR2, Anti- Hu, Ri, ANNA-3, Yo, Tr/DNER, Myelin, Ma/Ta, GAD65, Amphiphysin, Glutamate receptors (type AMPA), LGI1, ZIC4, DPPX, CARPVIII, Glycine receptors, mGluR1, mGluR5, GABA-a-receptors, Rho GTPase-activating protein 26, ITPR1, Homer 3, MOG, Recoverin, Neurochondrin, GluRD2, Flotillin-1/2, IgLON5, neurexin-3-alpha, ERC1, Sez6l2, AP3B2, Contactin 1, Neurofascin 155, Neurofascin 186, AT1A3, KCNA2 and Dopamine receptor 2

^**^Tested by Human TREM2 DUOSET ELISA, R&D Systems, Catalog Number: DY1828-05

Boldface parameters are pathological

CSF analyses showed normal basic parameters and according to degeneration markers an A-T-N + profile with increased *neurofilament light chain* (Table 1). A large panel of anti-neuronal autoantibodies was negative in cell-based assays and indirect immunofluorescence on murine brain sections. Electroencephalography (EEG) revealed no epileptiform abnormalities but an asymmetric frontotemporal dysrhythmia during rest, with a pathological slowing of the dominant alpha frequency towards the theta band, and increased delta and beta power (Fig. 2A, B, Appendix). Previous research showed abnormal alpha activity in dementia [9] and that alpha slowing predicted cognitive impairment [3].

**Fig. 2:**
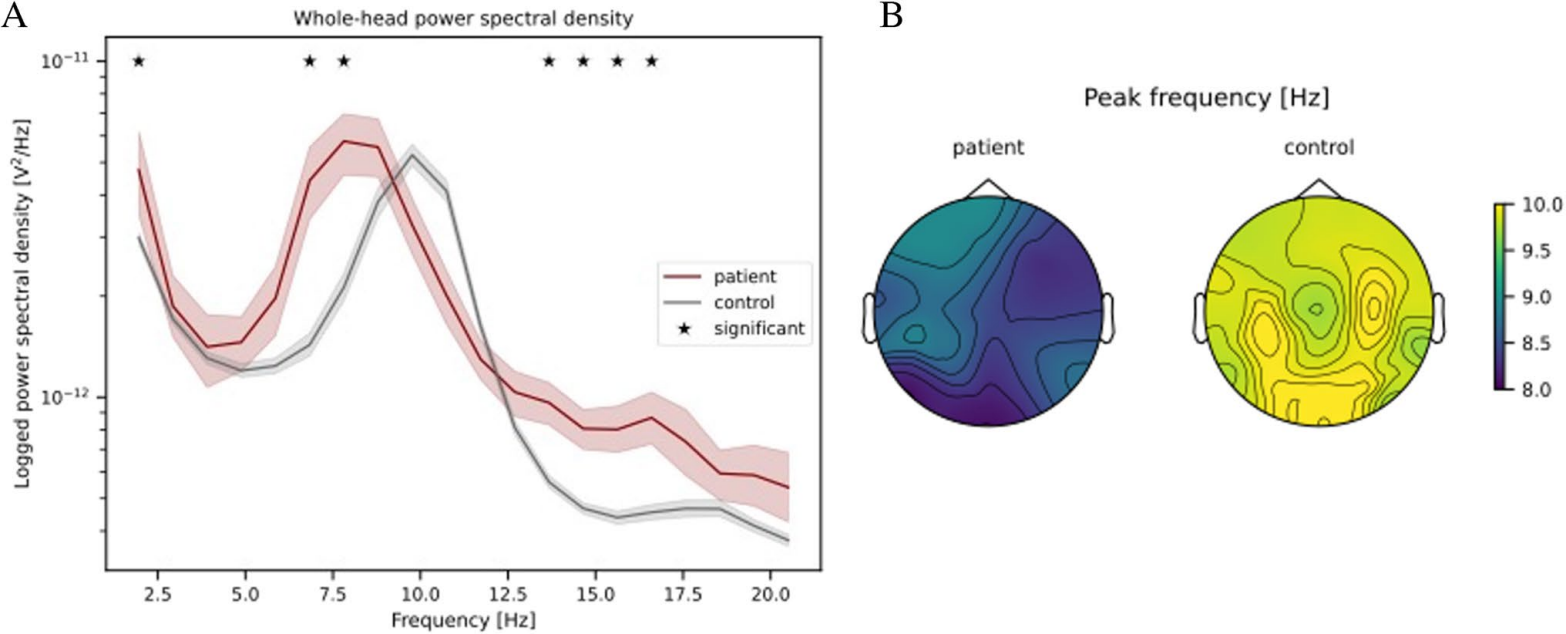
EEG findings are compatible with bvFTD. **A** The resting-state recording of the patient showed increased power spectral densities in the delta, theta/pre-alpha, and low beta band compared to healthy age and gender-matched controls (*n* = 15). Pixel-based permutations were performed to evaluate whole-head power differences across the frequency spectrum. Furthermore, the dominant frequency was lower in the patient (8.50 Hz) compared to healthy controls (*M* = 10.15 Hz, SD = 0.57 Hz) across all channels (*z* = 2.869, *p* = .002). **B** The topographical representation of peak frequencies across channels revealed an asymmetric frontotemporal dysrhythmia and generalized slowing of EEG activity

Following our memory clinic’s standard operating procedure, inconspicuous blood and CSF diagnostics was followed by genetic analyses using whole exome sequencing (WES) from the patient’s blood. WES identified two compound heterozygous mutations in the TREM2 gene, which in combination cause the clinical syndrome known as Nasu-Hakola disease (NHD) or polycystic lipomembranous osteodysplasia with sclerosing leukoencephalopathy (PLOSL). The variant c.313delG;p.Ala105Argfs*84 is already known from previous NHD cases [7], while the second mutation c199delC;pHis67Thrfs*9 has not been described so far, according to our knowledge. Genetic testing of both parents revealed one mutation in each of them, both were clinically unimpaired. The patient has two healthy younger half-siblings, who have not been tested so far. Upon re-evaluation of the ankle MRI, the constellation of several bone cysts filled with fatty components were considered indicative of lipomembranous osteodysplasia.

NHD/PLOSL is a rare disease (prevalence < 1:1.000.000) caused by loss-of-function mutations in the TREM2 or TYROBP genes [10]. The TREM2 gene is located on chromosome 6, the protein encoded by TREM2 is a transmembrane protein, acting as an activating cell surface receptor. The ectodomain is activated by ligand binding to initiate intracellular signalling pathways. It can independently be cleaved by different sheddases to a soluble form sTRME2 to regulate neuronal interactions and microenvironment [15]. Together with the transmembrane adaptor protein TYROBP the TREM2-receptor forms a complex to regulate the differentiation and function of osteoclasts [11]. Amongst several other functions, TREM2 plays an important role in activating macrophages and dendritic cells and is highly expressed in microglia [12]. The exact signalling pathways are poorly understood but seem to promote proliferation, phagocytosis, and migration of microglia by induction and maintenance of microglial activation [8]. Please check if the change is fine in this occurrence and modify the subsequent occurrences, if necessary.). Mutations in TREM2 are also associated with different forms of dementia, such as Alzheimer’s disease or FTD [14]. However, future research is needed to understand the pathogenic mechanisms of NHD/PLOSL, in particular the intracellular processes leading to cognitive impairment and brain dysfunction.

The clinical course of NHD/PLOSL can be divided into four stages [10]:

1. Latent stage: normal early development
2. Osseous stage (around 3rd life decade): pain and tenderness with a focus on ankles and feet, often followed by strain or injury
3. Early neurologic stage (around 4th decade of life): behavioural changes/ frontal lobe syndromes
4. Late neurologic stage: progressive dementia, loss of mobility and epileptic seizures

Our patient followed this clinical progression but developed stages 2–3 almost 10 years earlier than usual, possibly due to the combination of the here detected compound heterozygous TREM2 mutations. We assume that she will also reach stage 4 earlier, reflecting a rapidly progressing disease course.

We measured levels of sTREM2 in CSF and serum, which were significantly reduced compared to other dementia samples and healthy controls. Interestingly, the level of sTREM2 in CSF was not detectable at all (0.0 pg/ml) (see Table 1). This can be interpreted as a dysfunction of the remaining TREM2-receptors with impaired shedding of the TREM2-ectodomain, potentially also degradation of the mutated mRNA (nonsense-mediated decay) might occur, both resulting in sTREM2 isoform levels below the detection threshold.

Different genetic mutations that lead to NHD and partial or complete loss of function of TREM2 show decreased sTREM2 levels in CSF and come along with a decrease in shedding and ligand affinity [13].

Further management and care will focus on symptomatic treatments given the lack of effective causal therapies.

The present case demonstrates that low-threshold genetic testing in the neurological outpatient setting can definitely solve unclear constellations across the neuropsychiatric spectrum of symptoms and supersede years of uninstructive diagnostics. With higher availability of WES, the diagnosis in our patient could have been established already at the time of onset of psychiatric symptoms or even earlier at the time of orthopaedic management of bone cysts with characteristics of internal fatty tissue indicative for NHD. This would have markedly facilitated counselling of the patient and her family, especially in light of the serious behavioural symptoms. In our opinion, clinical judgement in such cases cannot substitute genetic testing, as some diseases are rare and established red flags, such as positive family history, disease-specific MRI changes or ‘typical’ symptoms are often absent or appear late in the disease course.

## Appendix

### EEG recording

EEG recordings were analyzed using custom-written code in Python. EEG resting-state activity was recorded for 10 min from 30 Ag/AgCl electrodes in the standard 10–20 international placement and mounted with the 32-channel waveguard^TM^ EEG cap (ANT Neuro, Hengelo, Netherlands). Impedances were kept below 10 kΩ. EEG was recorded with an eego^TM^ sports amplifier (ANT Neuro, Hengelo, Netherlands) and the respective software using a sampling rate of 500 Hz. Signal artifacts (e.g. related to eye blinks or head movements) were removed offline using an automated preprocessing pipeline to reduce bias possible with manual artefact rejection (MNE-Python package; [5). Slow drifts and line noise were removed with a 0.01-Hz high-pass filter and a 50-Hz notch filter, respectively. We excluded bad channels (*n*= 1) using the PREP pipeline [2] and replaced the data with interpolated signals recorded from surrounding electrode positions. The continuous EEG data were re-referenced to the common average and then band-pass filtered to 1–60 Hz and sliced into epochs of 2 s. Epochs which contained artefacts suggestive of eye blinks and other muscle activity were rejected based on maximum peak-to-peak signal amplitudes. We then computed estimations of the power spectral density in sensor space using Welch’s method and determined the peak frequency using the FOOOF-Python package [4]. Finally, results were compared with age and gender-matched EEG recordings of healthy volunteers (*n* = 15) taken from a publicly available EEG dataset [1].
